# Supplementary material for: Prevalence of depression and associated factors among obstetric care providers at public health facilities in the West Arsi Zone, Ethiopia: Cross-sectional study
Source: PLoS One. 2024 Jun 10;19(6):e0304903. doi: 10.1371/journal.pone.0304903 (PMC11164399; doi:10.1371/journal.pone.0304903)
Supplement: S1 Table — (DOCX) [file pone.0304903.s001.docx]

**Responses on depression assessment tool items on prevalence of depression among obstetric care providers in West Arsi zone, Ethiopia 2023**

| **S.no/** | **Categories** | **Response** | | | |
| --- | --- | --- | --- | --- | --- |
|  |  | **0(No symptom)** | **1(Mild symptom)** | **2 (Moderate symptom)** | **3 (severe symptom)** |
| 1 | Little interest or pleasure in doing things | 137(33%) | 165(39.8%) | 45(10.8%) | 68(16.4) |
| 2 | Feeling down, depressed, or hopeless | 31(7.5%) | 102(24.7%) | 85(20.3%) | 197(47.5) |
| 3 | Trouble falling or staying asleep, or sleeping too much | 205(49.  4%) | 103(24.8%) | 89(21.5%) | 18(4.3%) |
| 4 | Feeling tired or having little energy | 101(24.3%) | 58(14%) | 200(48.2%) | 56(13.5%) |
| 5 | Poor appetite or overeating | 96(23.1%) | 251(60.5%) | 25(6%) | 43%)(10.4%) |
| 6 | Feeling bad about yourself or that you are a failure | 88(21.1%) | 141(34%) | 117(28.2%) | 69(16.6%) |
| 7 | Trouble concentrating on things, such as reading the newspaper or watching television | 115(27.7%) | 203(48.9%) | 80(19.3%) | 17(4.1%) |
| 8 | Moving or speaking so slowly that other people could had noticed. Or the opposite being so | 164(39.5%) | 58(14%) | 144(34.7%) | 49(11.8%) |
| 9 | Thoughts that you would be better of dead, or of hurting yourself | 290(69.9%) | 92(22.2%) | 18(4.3%) | 15(3.6%) |

**Responses on burnout assessment tool items on prevalence of depression among obstetric care providers in West Arsi zone, Ethiopia 2023**

| **S.no/** | **Categories** | **Response** | | | |
| --- | --- | --- | --- | --- | --- |
|  |  | **0(Never)** | **1(Some time)** | **2(Often)** | **3(Always)** |
| 1 | Do you had irritability symptom in you work | 36(8.7%) | 156(37.6%) | 158(38.1%) | 65(15.7%) |
| 2 | Debility | 41(9.9%) | 134(32.3%) | 165(39.8%) | 75(18.1) |
| 3 | Self‑criticism | 75(18.1%) | 134(32.3%) | 165(39.8%) | 41(9.9%) |
| 4 | Insomnia | 145(34.9%) | 80(19.3%) | 135(32.5%) | 55(13.3%) |
| 5 | Fatigue | 158(38.1%) | 156(37.6%) | 36(8.7%) | 65(15.7%) |
| 6 | Spinal problem | 134(32.3%) | 42(10%) | 165(39.8%) | 74(18) |
| 7 | Lack of organization | 146(35.2%) | 73(17.6%) | 121(29.2%) | 75(18.1%) |
| 8 | Lack of sense of priority | 34(8.2%) | 187(45.1%) | 155(37.3%) | 39(9.4%) |
| 9 | Depressive states | 186(45%) | 34(8.2%) | 155(37.3%) | 40(9.5%) |
| 10 | Feeling of failure | 88(21.1%) | 141(34%) | 117(28.2%) | 69(16.6%) |
| 11 | Painful symptoms | 134(32.3%) | 189(45.5%) | 46(11.1%) | 46(11.1%) |
| 12 | Social isolation | 193(46.5%) | 114(27.5%) | 70(16.9%) | 38(9.2%) |
| 13 | Poor concentration and performance | 164(39.5%) | 144(34.7%) | 58(14%) | 49(11.8%) |
| 14 | Less caring attitude | 165(39.8%) | 86(20.7%) | 90(21.7%) | 74(17.8%) |
| 15 | Problem with rest of team | 49(11.8%) | 90(21.7%) | 87(21%) | 189(45.5%) |
| 16 | Dissatisfaction with work | 51(12.3%) | 205(49.4%) | 90(21.7%) | 96(16.6%) |
| 17 | Change of profession | 295(71.1%) | 47(11.3%) | 53(12.8%) | 20(4.8%) |
| 18 | Consume analgesics | 299(72%) | 85(20.1%) | 23(5.5%) | 8(1.9%) |
| 19 | Consume sedative | 325(78.3%) | 45(10.8%) | 30(7.3%) | 15(3.6%) |
