# Supplementary material for: Prevalence of depression and associated factors among obstetric care providers at public health facilities in the West Arsi Zone, Ethiopia: Cross-sectional study
Source: PLoS One. 2024 Jun 10;19(6):e0304903. doi: 10.1371/journal.pone.0304903 (PMC11164399; doi:10.1371/journal.pone.0304903)
Supplement: Attachment — Submitted filename: review.docx [file pone.0304903.s002.docx]

Thank you for invitation to review manuscript entitled as Prevalence of Depression and Associated Factors among Obstetric Care Providers: Cross-sectional study

And so the following comments are my concern regarding this paper.

**Title**

Why you didn’t include private health facilities (I need justification)

Why you include all obstetric care providers (it is too many) and I need justification.

**Abstract**

**Current substance use**

Do you think that this response is correct? Do you think that health care provider respond to this question in our setting???And if so what types of substance they have been used? Try to make it specify, because there are different types of substance

Having burnout symptoms (how did you measure it???)

**Methology**

It is better if you mention the name of hospital

Your study population is too many (midwives, gynecologist, nurse, integrated emergency surgical officers, HO) so why you include all of them???

Obstetrics care providers who have worked for at least six months were included? What is your justification?

It is better if you add the number of proportionally allocated individuals from each hospital

How did you approach and get your study population? I.e. have you got your study participants altogether?

**Study variable**

Occupation? Is not necessary. They are health care provider

Data collection tools

You used English version of quastionaries.so Do you think that all of your study participants equally understand English language, particularly in our setup???

**Data quality control**

Your data quality control is not detail explained?

**Recommendation**

Your recommendation need revision
