# Supplementary material for: Prevalence of depression and associated factors among obstetric care providers at public health facilities in the West Arsi Zone, Ethiopia: Cross-sectional study
Source: PLoS One. 2024 Jun 10;19(6):e0304903. doi: 10.1371/journal.pone.0304903 (PMC11164399; doi:10.1371/journal.pone.0304903)
Supplement: Attachment — Submitted filename: Response to Reviewers.docx [file pone.0304903.s003.docx]

**Response to Reviewers and Academic Editors**

- First of all, we sincerely appreciate your contribution to review of our work and your helpful suggestions. Based on AE and reviewers comments and suggestions we try to address each point as following: We also incorporated in revised document with track change and highlights.
- **Response to comments and suggestions raised by Academic Editor**

***Response:***

- Regarding the ***PLOS One journal guideline issue***, we corrected the revised document in accordance with the guideline.
- Regarding issues of ***data availability;*** previously we have stated that the current manuscript is a part of undergoing research project owned by Madda Walabu University Shashemenne Campus. All relevant data are included within the paper. The data would be guarded carefully by our research team for the only purpose of this scientific study and it is an ongoing project. For this reason and following the indicators of the research review committee of college of health sciences, Madda Walabu University, the authors must not upload the dataset to a stable, public repository. Interested, qualified researchers can access the data by requesting the following concerned bodies.

The research coordinators of Madda Walabu University Shashemenne Campus **Mr. Habtamu Jarso** can be contacted

1. Habtemu Jarso (BSc, MPH-E, Assis. Professor)
   Shashemene Campus, Madda Walabu University
   Institutional email: [habtemu.jarso@mwu.edu.et](mailto:habtemu.jarso@mwu.edu.et)
2. Corresponding author**; Solomon Seyife:** email: [SoleSeifa@gmail.com](mailto:SoleSeifa@gmail.com)

- Beside this all necessary files have been uploaded on initial submission on the track accordingly.
- The ***ethical statement's location*** was changed in accordance with your suggestion.

**2. Response to comments raised by first reviewer**

- Please insert the line number for writing the comment line by line and resubmit again?

***Response:***

- *We appreciate your feedback and have already incorporated it into the updated document.*

**3. Response to comments raised by second reviewer**

1. The manuscript didn’t abide with the submission guideline of the PONE journal. E.g., the abstract section.

***Response:***

- Thank you very much for your suggestion; On the basis of your suggestion, a correction has been made to the updated document.

2. The aim of the study and the scope of the recommendations are different.

***Response:***

- Thank you very much for your suggestion. Since our study is a single study, it is not advisable to recommend policy makers; instead, it should be in line with our findings. We therefore made a correction after reevaluating our findings.

***‘’*** *We recommend health professionals take care of themselves from substance use, just to live as exemplary for communities. Secondly, we recommend concerned stakeholders to establish mechanisms to improve job satisfaction and overcome burnout by creating different incentive mechanisms that increase staff income, increase the number of staff, provide different rewards, and conduct regular supervision of their problems. Finally, stakeholders and governments should make evidence-based decisions and ensure proper utilization of financial and human resources at health facilities.”*

3. Lack of consistency throughout the document i.e. is that depression or major depressive disorder?

***Response:***

our study is focused on depression. So, we carefully revised the document and made correction in revised document.

4. The background needs critical revision and the authors should revisit how to write an introduction that convince the reader containing all essential element and entailed flawed sentences E.g. “…by the 2030 year” in the introduction section.

***Response:***

- Thank you very much for your suggestion after revising this section we try to rewrite it for instance based on your recommendation we corrected line number 113.

‘’By 2030, depression symptoms are predicted to overtake other causes as the second largest contributor to disability-adjusted life years (DALYs).’’

5. The methods section lacks concise and details. In addition, the sampling technique is doubtable since the obstetric care providers have various levels E.g., Midwifery and Medical doctor. Likewise, the way the dependent variable is described, it fails to differentiate the use either ‘validated’ or ‘structured questionnaire’, this was witnessed by reference number 23. What was the type of the analysis since it is composite variable?

***Response:***

**Issue of methodology**

Having carefully reviewed the document, we made the necessary corrections.

- With respect to the ***sampling method***, refer to lines 156–172, as we modified the detailed sampling procedure based on your recommendations.

‘*’The total number of healthcare providers working at an obstetric unit in selected public health facilities, which was obtained from the zonal health bureau, was 708. From similar data obtained from zonal health office those obstetric care providers that serving selected public health facilities were categorized as 455 Midwifery professional, 108 Nursing, 13 IESO, 39 General practitioner, 89 Health officer and 2 Obstetrician. Before the selection of study participants, proportionate allocations to each health facility were made.* *Finally, from the list, the required sample sizes of 423 obstetric care providers were selected by simple random sampling techniques’’*. ***For further check the document.***

- Issue ***‘dependent variable’*** it fails to differentiate the use either ‘validated’ or ‘structured questionnaire’, this was witnessed by reference number 23

***Response:***

We have written explicitly about the tool, in particular the dependent variable, validity, and translation of questionnaires. Could you please revise line number 203-216 once? As you pointed out in reference number 23, we only provide the operational definition of the dependent variable; however, we make clear that about the tool in the Data Collection Tool and Procedure section.

- What was the type of the analysis since it is composite variable?

***Response:***

Based on the operational definitions of each variable we dichotomized those variables and proceed binary logistic regression. For instance for **dependent** variable. After adding each item score that used to assess depression. The total score of nine items ranging from 0 to 27. We dichotomized as score ≥ 10 were considered to have depression symptoms, and a code ‘1’ was given. While those who scored < 9 were considered to have no depression symptoms and a code‘0’. Other was also in similar fashion… then we run binary logistic regression and multivariable regression accordingly…….***some modification was made please refer the revised document.***

6. The results and the discussion are relatively better. But both should be presented concisely and completely in logical way. The discussion and the explanations are sometimes against evidence. E.g., HEWs demoralized.

***Response:***

Thank you very much for such constructive suggestion we made revision accordingly.

7. Individual roles of the authors should be explained independently and in detail

***Response:***

We corrected according to the guide line of PLOS one

‘’ SSA, MH, ZA, GMT, YAW, WG, MAH, ADG, ShMT and LFW have contributed equally to the proposal development, development of the tool, data collection process, and analysis. All the authors have revised the final version of the manuscript and given their approval for publication.’’

8.  Poor referencing E.g., Reference number 5.

***Response:***

Frankly speaking we use some of references direct from web site of different organization that is why some references have lack of full information. However, as much as possible after revision we made a correction accordingly. For instance based on your suggestion reference number 5; we add its URL; Mind Vw. Mood Swings: Causes, Risk Factors, and Ways to Cope. Dotdash Media. 2023. (<https://www.verywellmind.com/what-are-mood-swings-1067178>)

**Concerns about this paper were addressed as follow:**

1. **Title**
A. Why you didn’t include private health facilities (I need justification)

***Response:***

- Due to the fact that most private health facilities do not offer all obstetric and gynecological procedures, we only included governmental facilities. For example, labor and delivery service. Another reason is that the majority of healthcare providers who work in public healthcare facilities are also doing so in the private sector. Finally, the researcher's area of interest is depression among obstetric care providers who work in government health facilities, which have different challenges than private health institutions in terms of work load, income, and other factors.

B. Why you include all obstetric care providers (it is too many) and I need justification.

***Response:***

- We encompass all obstetric care providers because we have to deal with the working environment in which they carry a comparatively similar burden. Furthermore, depression affects everyone equally and is not selective.

2. Current substance use Do you think that this response is correct? Do you think that health care provider respond to this question in our setting???And if so what types of substance they have been used? Try to make it specify, because there are different types of substance

***Response:***

- We appreciate you raising these questions. Though social desirability bias plays a part in the issue, all relevant information had been given prior to data collection, so we hope that they were fully provided with information in accordance.
- Actually, during the process of collecting data our questionnaires, addressed commonly used substance such as alcohol, cigarettes, and Kchat.

3. Having burnout symptoms (how did you measure it???)

**Please refer line number 193-197**

**A burnout symptom was measured by previously validated tool in Ethiopia.**

***Response:***

**‘’Burnout:** There are 19 items to assess symptoms of burnout syndrome (19 items) that are categorized into four Likert scales 0 (never), 1 (sometimes), 2 (often), and 3 (always). The sums of the scores of the rating of the items were calculated with a total minimum score of 0 to a maximum score of Healthcare. Obstetrics care providers that scored more than 23 out of 57 were considered to have burnout symptoms (4).’’

4. Methology
A. It is better if you mention the name of hospital

Your study population is too many (midwives, gynecologist, nurse, integrated emergency surgical officers, HO) so why you include all of them??? It is better if you add the number of proportionally allocated individuals from each hospital

***Response:***

- Based on your recommendations we try to narrate accordingly on revised document. For schematic presentation due to many (37) health facilities it’s difficult to put it in one diagram. So to handle this issue we try to explain in narrative form and just it’s self-explanatory; please refer sampling technique procedure. If must we will put it…

B. Obstetrics care providers who have worked for at least six months were included? What is your justification?

***Response:***

- We exclude those health providers due to finally we conclude this study for that study setting. Secondly, those health care providers were not fully engaged in working environments.

5. Study variable Occupation? Is not necessary. They are health care provider

***Response:***

Thanks for your correction we accept it and already removed.

6. Data collection tools

You used English version of quastionaries.so Do you think that all of your study participants equally understand English language, particularly in our setup???

***Response:***

- Thanks for your suggestion but we clearly stated in manuscript as following: please check line number 212-214

‘’The questionnaire was prepared in English and later translated into Afan Oromo to check the consistence of questionnaire, and English language was used for data collection’’

7. Data quality control

Your data quality control is not detail explained?

***Response:***

We can add some important points. Refer from revised document

8. Recommendation

Your recommendation need revision

***Response:***

- Thank you very much for your suggestion. Since our study is a single study, it is not advisable to recommend policy makers; instead, it should be in line with our findings. We therefore made a correction after reevaluating our findings.
