# Supplementary material for: Prevalence of depression and associated factors among obstetric care providers at public health facilities in the West Arsi Zone, Ethiopia: Cross-sectional study
Source: PLoS One. 2024 Jun 10;19(6):e0304903. doi: 10.1371/journal.pone.0304903 (PMC11164399; doi:10.1371/journal.pone.0304903)
Supplement: Attachment — Submitted filename: Response to Reviewers.docx [file pone.0304903.s004.docx]

**Response to Reviewers and Academic Editors**

- First of all, we sincerely appreciate your contribution to review of our work and your helpful suggestions. Secondly we appreciate AE and all reviewers for re-revised our document and put your constructive suggestions and corrections. Based on AE and reviewers comments and suggestions we try to address each point as following: We also incorporated in revised document with track change and highlights. We prepared revised documents in both track change and clear documents based on the journal guide lines.

***Journal Requirements:*** *Please review your reference list to ensure that it is complete and correct. If you have cited papers that have been retracted, please include the rationale for doing so in the manuscript text, or remove these references and replace them with relevant current references. Any changes to the reference list should be mentioned in the rebuttal letter that accompanies your revised manuscript. If you need to cite a retracted article, indicate the article’s retracted status in the References list and also include a citation and full reference for the retraction notice.*

***Response:***

- We really appreciate your suggestions. Based on your recommendations, we thoroughly checked all references, and we didn’t find any retracted references. We also kindly request that if you get any retracted references, indicate the reference, and we are ready to make corrections at any time during this review period. Thank you very much again.

**Response to reviewer -3**

- *Try to abide the comments given before. Conclusion and recommendation section should be revised. Some grammatical errors should be corrected*

***Response:***

- Thank you very much for your constructive suggestion. We take note of the feedback and have included it into the updated document.

**Response to reviewer -4**

1. *There is not clear justification for the choice of the variable tested for religion and ethnicity. How do they contribute to the depression? Authors should explain to the reader how/why these variables are important. This should be clearly outlined. For example the salary can have an impact on depression duel to the global economic challenges we are witnessing today.*

***Response:***

- We sincerely appreciate your kind inquiry. You provide a clear explanation of the factors of ethnicity and religion. We also recognize that it can be challenging to link those factors to various illnesses, complications, and other health issues. We only included the descriptive portion and did not perform any association analysis with depression for those variables. We included those variables in the descriptive table solely to describe the sociodemographic characteristics of the population under study.

2. *Can the authors explain the impact of the different seen in the frequencies for example in the “Types of health providers”. In that section we only have 2 obstetricians and 311 midwifery. Statistically there will be difference and that this mean that obstetricians are not depressed? The appear to be a problem in the frequencies and this has a chance of obscuring the “true” in the analysis and interpretation.*

***Response:***

- Thank you very much for your question. From the beginning, our study was among obstetric care providers, meaning all types of health care providers who serve in obstetrics and gynecology wards. Initially, we obtain the numbers and types of health care providers that serve Obs/Gyn in selected institutions from the zonal health office. 2 obstetricians and 311 midwives were selected as health professionals during the selection process by simple random sampling techniques from all obstetric care providers found in selected institutions.

3. *In line 356 in the revised manuscript, the number from Vietnam are missing….this information should be provided and the source cited.*

***Response:***

- Thank you very much. We take note of the feedback and have included it into the updated document.

4. *In line 360 of the revised manuscript, the authors should cite the studies conducted in this countries.*

***Response:***

- Thank you very much. We take note of the feedback and have included it into the updated document.

*5. From line 370-375; the authors discuss the effect of working hours and those working over 40 hrs a week appear to be more depressed. What did they observe in the individuals working <40 hrs a week? That need to be articulated in the discussion.*

- ***Response:*** Thank you very much for your suggestions. We accepted and incorporated it in the revised document.

***‘’In addition, working extra limits an individual's free time for relaxation, leading to exhaustion and poor sleep, a combination that might lead to depression. On the other hand, having enough time is crucial for better relaxation, which boosts mood, reduces stress, and improves memory and focus. These benefits enable him or her to return to work with a renewed mindset.’’***

*6. Response: “We recommend health professionals take care of themselves from substance use, just to live as exemplary for communities.” Should read: We recommend health professionals take care of themselves and avoid substance use”*

***Response:***

- Thank you very much for your suggestions. We accepted and incorporated it in the revised document.

*7. The data used in this study should be anonymized and made available in the public spaces such as FigShare before the work is published.*

***Response: Thank very much for your suggestions.***

**Availability of data and materials**

- This current manuscript is a part of undergoing research project owned by Madda Walabu University Shashemenne Campus. All relevant data are included within the paper. The data would be guarded carefully by our research team for the only purpose of this scientific study and it is an ongoing project. For this reason and following the indicators of the research review committee of college of health sciences, Madda Walabu University, the authors must not upload the dataset to a stable, public repository. Interested, qualified researchers can access the data by requesting the following concerned bodies.

The research coordinators of Madda Walabu University Shashemenne Campus **Mr. Habtamu Jarso** can be contacted

1. Habtemu Jarso (BSc, MPH-E, Assis. Professor)
   Shashemene Campus, Madda Walabu University
   Institutional email: [habtemu.jarso@mwu.edu.et](mailto:habtemu.jarso@mwu.edu.et)

Corresponding author**; Solomon Seyife:** Email: [SoleSeifa@gmail.com](mailto:SoleSeifa@gmail.com)

**Response to reviewer -5**

*1. I suggest the authors include the setting in the title
Example: Prevalence of Depression and Associated Factors among Obstetric Care Providers at public health facilities in the West Arsi Zone, Ethiopia: Cross-sectional study*

***Response:*** Thank you very much for your constructive suggestion. We accepted your recommendations and incorporated in to revised document.

*2. I request the authors to add a supplementary table with the individual responses (frequencies and percentages) to each of the items in the following: The Patient Health Questionnaire-9, burnout 19 items, and The OSSS-3 sum score.*

***Response:***

- We really appreciate your request. We incorporated your request as supplementary tables. You can check it out. We provide the title “Supplementary Table.”

**Thank you very much again for the scientific suggestion.**
